# Supplementary figures and images for: MicroRNA Tissue Atlas of the Malaria Mosquito Anopheles gambiae
Source: G3 (Bethesda). 2017 Nov 16;8(1):185–93. doi: 10.1534/g3.117.300170 (PMC5765347; doi:10.1534/g3.117.300170)

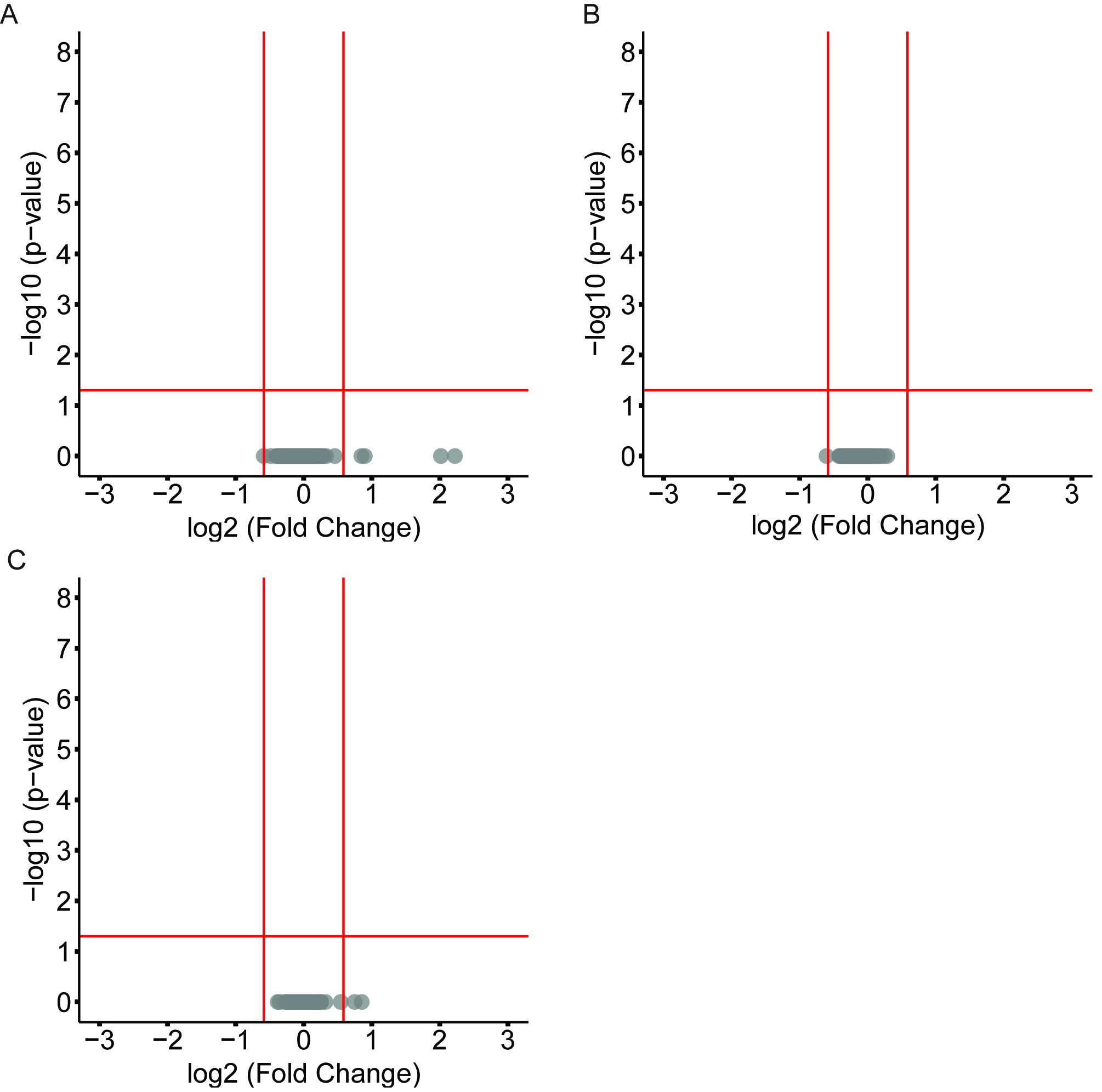

Supplement: Supplementary file 1 [file 185FigureS1.tif]

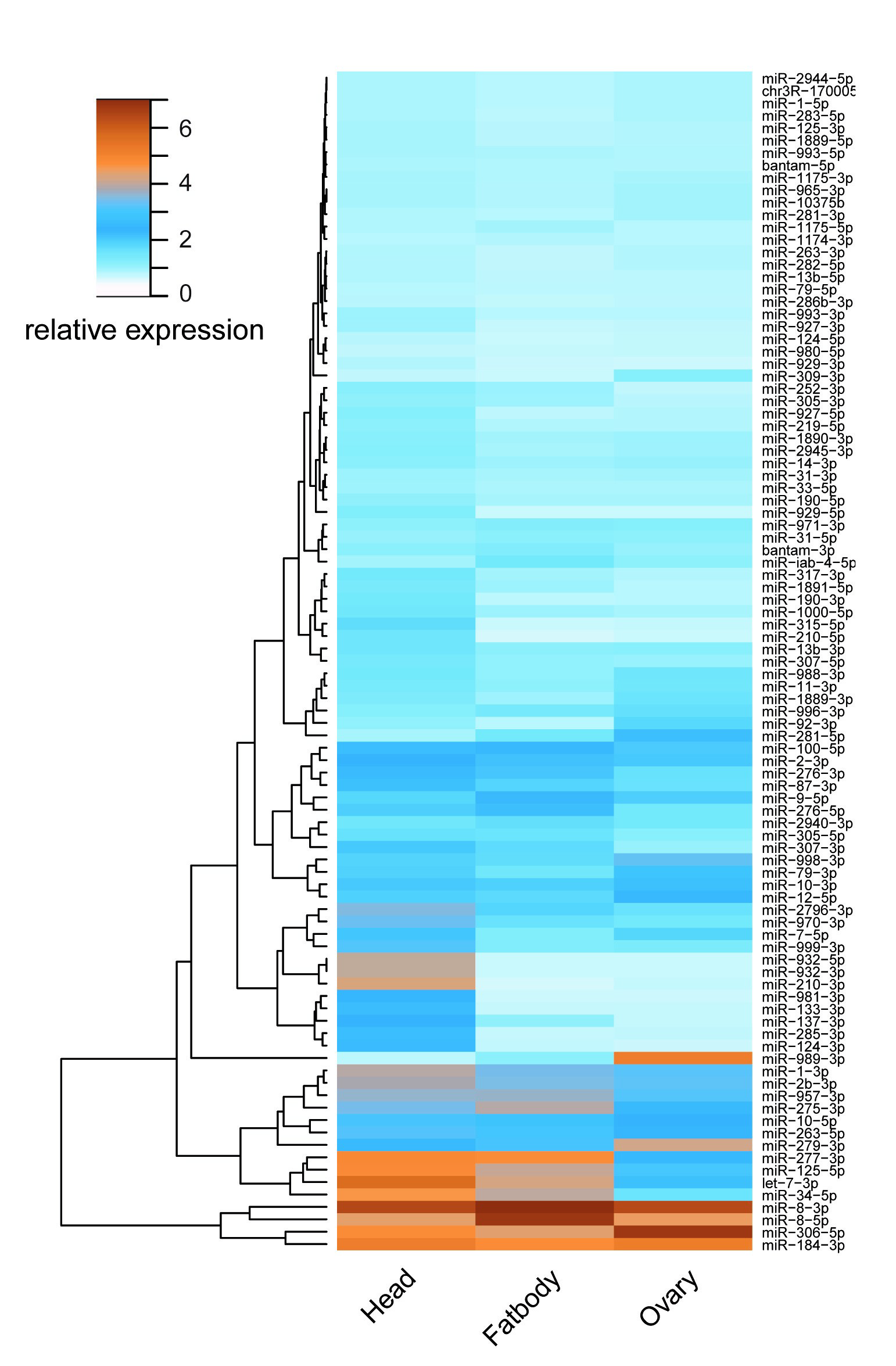

Supplement: Supplementary file 2 [file 185FigureS2.tif]
