# Supplementary material for: MicroRNA Tissue Atlas of the Malaria Mosquito Anopheles gambiae
Source: G3 (Bethesda). 2017 Nov 16;8(1):185–93. doi: 10.1534/g3.117.300170 (PMC5765347; doi:10.1534/g3.117.300170)
Supplement: Supplementary file 3 [file 185FileS1.docx]

Supplemental Figure Legends

**Figure S1: Tissue-specific miRNA expression in blood-fed A. gambiae.** Heatmap of miRNA expression levels in the head, midgut, fat body and ovaries at 18 h post blood meal. Colour gradient from light blue to dark brown represents the increase in miRNA expression.

**Figure S2: Differentially expressed miRNAs at 18 h post P. falciparum-infected blood feeding in A. gambiae tissues.** (A) fat body, (B) ovaries and (C) head. Cut-off log10 p-value of +/-1.3 (p<0.05) and log2 fold change of +/-0.58 (FC>1.5) (n=4) were used to detect significant changes in miRNA expression by fitting a linear model with empirical bayes statistics.

Supplemental Tables

Table S1: The microarray repertoire of A. gambiae miRNA arms

| aga-miR-212 | aga-miR-79-5p | aga-miR-278-3p | |
| --- | --- | --- | --- |
| aga-miR-929-5p | aga-miR-87-3p | aga-miR-9c-5p | |
| aga-miR-100-5p | aga-mir-2796-5p | aga-miR-9a-5p | |
| aga-miR-281-5p | aga-mir-2940-5p | aga-miR-957-3p | |
| aga-miR-1175-5p | aga-mir-33-3p | aga-miR-1890-3p | |
| aga-miR-308-3p | aga-miR-263b-3p | aga-miR-317-3p | |
| aga-miR-282-5p | aga-miR-1174-5p | aga-mir-286b-5p | |
| aga-miR-92b-3p | aga-miR-988-5p | aga-mir-2945-5p | |
| aga-miR-125-3p | aga-miR-79-3p | aga-miR-279-3p | |
| aga-miR-1889-3p | aga-miR-9c-3p | aga-miR-996-3p | |
| aga-mir-10368-5p | aga-miR-283-5p | aga-bantam-3p | |
| aga-miR-10-5p | aga-miR-277-3p | aga-miR-1175-3p | |
| aga-miR-iab-4-5p | aga-mir-10375b-3p | aga-miR-1891-5p | |
| aga-miR-307-5p | aga-miR-965 | aga-let-7-5p | |
| aga-miR-9b-5p | aga-miR-965-3p | aga-miR-12-5p | |
| aga-miR-190-5p | aga-miR-124-3p | aga-miR-219-5p | |
| aga-miR-276-5p | aga-miR-8-3p | aga-mir-932-3p | |
| aga-miR-210-5p | aga-miR-993-5p | aga-mir-2c-3p | |
| miR-iab-8-3p | aga-mir-998-5p | aga-miR-7-5p | |
| aga-mir-10355-3p | aga-mir-285-5p | aga-miR-1-3p | |
| aga-miR-1000-5p | aga-mir-31-3p | aga-miR-184-3p | |
| aga-miR-305-5p | aga-mir-980-3p | aga-mir-31-5p | |
| aga-miR-927-3p | chr2R_3959-miR* | aga-miR-72-1 | |
| aga-miR-10-3p | aga-miR-276-3p | aga-miR-34-5p | |
| aga-miR-1889-5p | chr2L_3426-miR | aga-miR-263a-5p | |
| aga-miR-11-3p | aga-mir-2a-1-3p | aga-miR-281-3p | |
| aga-miR-8-5p | aga-miR-2-1-3p | chrX_40848 | |
| aga-miR-1-5p | aga-miR-13b-3p | aga-chrX_353716 | |
| aga-miR-190-3p | aga-mir-2944a-1-3p | aga-miR-989-3p | |
| aga-bantam-5p | aga-miR-92a-3p | aga-mir-999-5p | |
| aga-miR-305-3p | aga-miR-137-3p | chrUNKN_30285 | |
| aga-miR-iab-4-3p | aga-mir-932-5p | chr2R_12239-miR | |
| aga-mir-980-5p | aga-miR-307-3p | aga-miR-981-3p | |
| aga-miR-263a-3p | aga-miR-309-3p | aga-miR-133-3p | |
| aga-miR-929-3p | aga-miR-3-1-miR | aga-miR-210-3p | |
| aga-mir-252-3p | aga-miR-1174-3p | aga-miR-927-5p | |
| aga-miR-263b-5p | aga-miR-275-3p | aga-miR-375-1-3p | |
| aga-miR-315-3p | aga-miR-306-5p | aga-miR-315-5p | |
| aga-miR-993-3p | aga-miR-14-3p |  |  |
| aga-mir-2944b-5p | aga-miR-970-3p |  |  |
| aga-mir-2944a-5p | aga-miR-125-5p |  |  |

Table S2: Proportion of miR-3p arm expression of miRNAs expressing both miRNA arms.

| **miRNA** | **Fat body** | **Head** | **Ovary** | **Midgut** | **Standard deviation** |
| --- | --- | --- | --- | --- | --- |
| bantam | 0,744 | 0,575 | 0,630 | 0,515 | 0,097 |
| miR-1 | 0,950 | 0,933 | 0,960 | 0,917 | 0,019 |
| miR-10 | 0,761 | 0,611 | 0,799 | 0,605 | 0,101 |
| miR-1175 | 0,468 | 0,549 | 0,537 | 0,285 | 0,122 |
| miR-124 | 0,240 | 0,855 | 0,465 | 0,440 | 0,257 |
| miR-125 | 0,104 | 0,108 | 0,082 | 0,115 | 0,014 |
| miR-13 | 0,665 | 0,644 | 0,766 | 0,709 | 0,054 |
| miR-190 | 0,511 | 0,560 | 0,385 | 0,517 | 0,075 |
| miR-210 | 0,691 | 0,765 | 0,519 | 0,449 | 0,147 |
| miR-263 | 0,130 | 0,223 | 0,152 | 0,444 | 0,144 |
| miR-276 | 0,502 | 0,599 | 0,674 | 0,522 | 0,079 |
| miR-281 | 0,308 | 0,539 | 0,159 | 0,174 | 0,176 |
| miR-305 | 0,423 | 0,333 | 0,362 | 0,423 | 0,045 |
| miR-307 | 0,700 | 0,605 | 0,530 | 0,443 | 0,109 |
| miR-31 | 0,328 | 0,526 | 0,340 | 0,391 | 0,091 |
| miR-79 | 0,718 | 0,831 | 0,916 | 0,820 | 0,081 |
| miR-8 | 0,557 | 0,606 | 0,608 | 0,561 | 0,028 |
| miR-927 | 0,326 | 0,398 | 0,411 | 0,438 | 0,048 |
| miR-929 | 0,188 | 0,344 | 0,520 | 0,492 | 0,153 |
| miR-993 | 0,623 | 0,514 | 0,478 | 0,514 | 0,063 |

Table S3: One-way ANOVA results for the differential expression of miRNA arm proportion across tissues.

| **miRNA** | **One-way ANOVA (p-value)** |
| --- | --- |
| miR-10 | 0.0244 |
| miR-1175 | 0.00395 |
| miR-124 | 9.18e-07 |
| miR-210 | 1.7e-05 |
| miR-263 | 9.76e-05 |
| miR-281 | 2.7e-05 |
| miR-307 | 1.65e-05 |

Table S4: Description of the microarray data files submitted to GEO expression omnibus.

| File number | File name | Mosquito tissue | Experimental  Condition | Time point |
| --- | --- | --- | --- | --- |
| \| 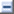GSM2752776 \| \| --- \| | UF_Carcass_1 | Abdominal carcass | Sugar fed | respective TP to 18 hpb |
| GSM2752777 | UF_Carcass_2 | Abdominal carcass | Sugar fed | respective TP to 18 hpb |
| GSM2752778 | UF_Carcass_3 | Abdominal carcass | Sugar fed | respective TP to 18 hpb |
| GSM2752779 | UF_Carcass_4 | Abdominal carcass | Sugar fed | respective TP to 18 hpb |
| GSM2752780 | UF_Midgut_1 | Midgut | Sugar fed | respective TP to 18 hpb |
| GSM2752781 | UF_Midgut_2 | Midgut | Sugar fed | respective TP to 18 hpb |
| GSM2752782 | UF_Midgut_3 | Midgut | Sugar fed | respective TP to 18 hpb |
| GSM2752783 | UF_Midgut_4 | Midgut | Sugar fed | respective TP to 18 hpb |
| GSM2752784 | UF_Ovaries_1 | Ovaries | Sugar fed | respective TP to 18 hpb |
| GSM2752785 | UF_Ovaries_2 | Ovaries | Sugar fed | respective TP to 18 hpb |
| GSM2752786 | UF_Ovaries_3 | Ovaries | Sugar fed | respective TP to 18 hpb |
| GSM2752787 | UF_Ovaries_4 | Ovaries | Sugar fed | respective TP to 18 hpb |
| GSM2752788 | UF_Head_1 | Head | Sugar fed | respective TP to 18 hpb |
| GSM2752789 | UF_Head_2 | Head | Sugar fed | respective TP to 18 hpb |
| GSM2752790 | UF_Head_3 | Head | Sugar fed | respective TP to 18 hpb |
| GSM2752791 | UF_Head_4 | Head | Sugar fed | respective TP to 18 hpb |
| GSM2752792 | UI_Carcass_1 | Abdominal carcass | human blood | 18 hpb |
| GSM2752793 | UI_Carcass_2 | Abdominal carcass | human blood | 18 hpb |
| GSM2752794 | UI_Carcass_3 | Abdominal carcass | human blood | 18 hpb |
| GSM2752795 | UI_Carcass_4 | Abdominal carcass | human blood | 18 hpb |
| GSM2752796 | UI_Ovaries_1 | Ovaries | human blood | 18 hpb |
| GSM2752797 | UI_Ovaries_2 | Ovaries | human blood | 18 hpb |
| GSM2752798 | UI_Ovaries_3 | Ovaries | human blood | 18 hpb |
| GSM2752799 | UI_Ovaries_4 | Ovaries | human blood | 18 hpb |
| GSM2752800 | UI_Head_1 | Head | human blood | 18 hpb |
| GSM2752801 | UI_Head_2 | Head | human blood | 18 hpb |
| GSM2752802 | UI_Head_3 | Head | human blood | 18 hpb |
| GSM2752803 | UI_Head_4 | Head | human blood | 18 hpb |
| GSM2752804 | I_Carcass_1 | Abdominal carcass | *P. falciparum*-infected human blood | 18 hpb |
| GSM2752805 | I_Carcass_2 | Abdominal carcass | *P. falciparum*-infected human blood | 18 hpb |
| GSM2752806 | I_Carcass_3 | Abdominal carcass | *P. falciparum*-infected human blood | 18 hpb |
| GSM2752807 | I_Carcass_4 | Abdominal carcass | *P. falciparum*-infected human blood | 18 hpb |
| GSM2752808 | I_Ovaries_1 | Ovaries | *P. falciparum*-infected human blood | 18 hpb |
| GSM2752809 | I_Ovaries_2 | Ovaries | *P. falciparum*-infected human blood | 18 hpb |
| GSM2752810 | I_Ovaries_3 | Ovaries | *P. falciparum*-infected human blood | 18 hpb |
| GSM2752811 | I_Ovaries_4 | Ovaries | *P. falciparum*-infected human blood | 18 hpb |
| GSM2752812 | I_Head_1 | Head | *P. falciparum*-infected human blood | 18 hpb |
| GSM2752813 | I_Head_2 | Head | *P. falciparum*-infected human blood | 18 hpb |
| GSM2752814 | I_Head_3 | Head | *P. falciparum*-infected human blood | 18 hpb |
| GSM2752815 | I_Head_4 | Head | *P. falciparum*-infected human blood | 18 hpb |

* TP = Time point; BM = blood meal; hpb = hours post blood feeding
